# Multi-model deep learning for dementia detection: addressing data and model limitations

**DOI:** 10.3389/fnins.2025.1638022

**Published:** 2025-11-25

**Authors:** Areej Y. Bayahya, Fares Jammal, Haneen Banjar, Fathy Elbouraey Eassa, Omar Talabay, Sultan H. Alamri

**Affiliations:** 1 Computer Science Department, Faculty of Computing and Information Technology, King Abdulaziz University, Jeddah, Saudi Arabia; 2 Software Engineering Department, College of Engineering, University of Business and Technology (UBT), Jeddah, Saudi Arabia; 3 Department of Computer Science, Faculty of Computing and Information Technology, King Abdulaziz University, Rabigh, Saudi Arabia; 4 Center of Research Excellence in Artificial Intelligence and Data Science, King Abdulaziz University, Jeddah, Saudi Arabia; 5 Institute of Genomic Medicine Sciences, King Abdulaziz University, Jeddah, Saudi Arabia; 6 Centre of Artificial Intelligence in Precision Medicines, King Abdulaziz University, Jeddah, Saudi Arabia; 7 Future Artificial Intelligence Company (Humain), Riyadh, Saudi Arabia; 8 Department of Family Medicine, Faculty of Medicine, King Abdulaziz University, Jeddah, Saudi Arabia

**Keywords:** 3D-CNN, AI, dementia, deep neural network, caps network, ViT, CNNs, XAI

## Abstract

**Introduction:**

Deep neural network architectures have transformed medical imaging, particularly in structural MRI (sMRI) classification. However, existing state-of-the-art deep learning models face limitations in preprocessing and feature extraction when classifying dementia-related conditions. This study addresses these challenges by evaluating multiple architectures for dementia diagnosis.

**Methods:**

This study assessed eight pretrained convolutional neural networks (CNNs), a Vision Transformer (ViT), a multimodal attention model, and a capsule network (CapsNet) for classifying three classes: dementia, mild cognitive impairment (MCI), and healthy controls. The dataset, obtained from ADNI, was balanced across classes and comprised 10,000 training images per class, 3,000 validation images per class, and 850 test images per class. Classification was performed using 2D slices from sMRI scans. Performance metrics included accuracy, specificity, and sensitivity.

**Results:**

Among all evaluated models, the 3D-CNN and multimodal attention models achieved the highest performance, with accuracies of 84% and 86%, specificities of 83% and 86%, and sensitivities of 84% and 86%, respectively. The ViT and CapsNet models achieved 100% sensitivity for Alzheimer’s disease (AD) but demonstrated low precision for AD (43%) and 0% for other classes, indicating class imbalance effects. All models showed reduced performance and bias toward certain classes.

**Discussion:**

The findings highlight the limitations of current architectures in sMRI dementia classification, including suboptimal feature extraction and class-specific biases. While certain models, such as multimodal attention and 3D-CNN, performed better overall, precision and generalization remain challenges. Future work should focus on improved data representation through advanced computer vision methods and architectural modifications to enhance diagnostic accuracy and computational efficiency.

## 1 Introduction

Multiple deep neural network architectures have fundamentally transformed the domain of medical imaging, presenting unprecedented capabilities for the classification in Magnetic Resonance Imaging (MRI) (Lecun et al., 2015). In the context of neuroimaging, the identification of structural anomalies, particularly those associated with conditions such as Mild Cognitive Impairment (MCI), requires the employment of models that can handle the intricate, overlapping, and frequently subtle distinctions in brain structures (Bayahya et al., 2021).

Despite the prevalent dominance of traditional models such as convolutional neural networks (CNNs) and their variations in medical image investigation filed, a notable insight is that the specific architectural design is not the primary factor in attaining optimal performance outcomes. For instance, in challenges like MCI, numerous researchers utilize the same architectural framework but obtain different outcomes. A frequently disregarded consideration that domain-specific expertise is related to the task at hand can provide significant advantages beyond simply augmenting the number of layers within a CNN (Litjens et al., 2017). Despite that CNNs is better at capturing local spatial details acquisition owing to their hierarchical architecture and has ability to learn features autonomously, negating the necessity for prior domain knowledge or manual segmentation (Litjens et al., 2017). However, CNNs fall short due to the complexity of the data, the variability inherent in imaging protocols, and the necessity for elevated sensitivity and specificity (Lecun et al., 2015).

Recent advancements in artificial intelligence (AI) have introduced promising deep learning architectures, such like Vision Transformer (ViT) and Capsule Networks (CapsNets) for medical imaging classification. However, these models remain largely unexamined for dementia diagnosis using sMRI. CapsNets have not yet been implemented to sMRI-based dementia diagnosis. Although ViT has demonstrated across various medical imaging. However, it is conspicuous absence of studies that have applied specifically to sMRI-based dementia classification.

This study claims that classification of dementia using deep learning requires more sophisticated integration methods and preprocessing techniques, such as focusing on Regions of Interest (ROI) with advanced preprocessing techniques. Traditional batch processing architectures may not efficiently capture fine-grained patterns in medical imaging data, necessitating an improved approach for feature extraction and classification. This study investigates CapsNets and ViT to examine new possibilities for leveraging global context in brain imaging. Additionally, this study examined CNN models to evaluate and classify sMRI images for dementia patients. Based on high-performance criteria of pretrained CNN models, this study selected 8 out 20 models and elicited from a different family of CNN. In addition, it selected baseline and 3dCNN to compare with pretrained models. Finally, it applied multimodal attention model to enhance accuracy. This study focuses on evaluating the accuracy, F1, Precision, specificity and sensitivity of Dementia. The objectives of this study are:

- To develop a multi-model deep learning AI framework that integrates CNNs, Vision Transformers, multimodal attention, and Capsule Networks for sMRI image classification in dementia detection.
- To explore and compare multiple CNN families and select the best-performing pre-trained models from each family.
- To assess the impact of CNN families, ViT, multimodal attention and CapsNets on sMRI classification for demented and MCI patients.
- To classify patients into MCI, demented and healthy control categories using CNN family models, ViT, multimodal attention and CapsNets.
- To evaluate each model based on performance metrics, including sensitivity, accuracy, specificity, F1, Precision and others for each model.
- To investigate whether these models can achieve high performance without applying additional computer vision techniques or argumentation factors.

This article presents the Literature Review, Methodology, Performance Evaluation and Discussion of Results, followed by the Conclusion and References. Each section provides a structured analysis, ensuring a comprehensive understanding of the research.

## 2 Literature review

Recent advancements in AI and machine learning ML have been propelled by new neural network architectures. Among these, Convolutional Neural Networks have become fundamental in computer vision. It excels in image classification, object detection, and segmentation due to their ability to effectively capture spatial hierarchies in visual data. The hierarchical structure of CNNs allows for automatic feature extraction, essential for handling high-dimensional inputs.

The core components of a CNN encompass convolutional layers, pooling layers, nonlinear activation layers, and fully connected layers. Typically, an image undergoes preprocessing prior to fed into the network via the input layer. Subsequently, it is processed through a sequence of alternately arranged convolutional and pooling layers, culminating in classification using fully connected layers (Chen et al., 2021; Zhang et al., 2019; Zhang, 2019).

Additionally, ViT and CapsNet are new models that represent a paradigm shift in visual recognition, challenging the dominance of CNNs. Capsule Networks offer an innovative strategy to address the shortcomings of CNNs. It preserves the spatial hierarchies and handles the viewpoint variations. It constitutes a type of deep neural network engineered to capture complex spatial hierarchies and interrelations within data. It enhances image recognition and segmentation. Unlike CNN, CapsNets use capsules as vectors that encapsulate attributes such as size, position, and orientation of entities within an image (Chen et al., 2021; Zhang et al., 2019). ViT is an advanced architecture dedicated to image classification. ViT architecture employs self-attention mechanisms to capture global relationships across image patches (Bazi et al., 2021). It consists of an embedding layer, an encoder, and a classifier head. The model processes input images by partitioning them into non-overlapping patches, where each patch is treated as a token.

A lot of studies investigate the use of MRI brain scans in the identification and diagnosis of dementia. Dementia is delineated by severe symptoms such as memory impairment and cognitive decline it affects more than 47 million people worldwide with estimations anticipated 131 million by 2050, as reported in the World Alzheimer Report 2016.

The early diagnosis of MCI is crucial as it facilitates the recognition of those at risk for Alzheimer’s disease (AD) and leads to more effective treatment, thereby potentially postponing the progression of the disease. To address these issues, numerous investigations have employed deep neural networks such as (Carcagnì et al., 2023; Herzog and Magoulas, 2021; Qiu et al., 2022; Luo et al., 2017; Murugan et al., 2021; Taheri gorji and Kaabouch, 2019; Torghabeh et al., 2023; Li et al., 2019; Tomassini et al., 2023; Mohammed et al., 2021; Sai et al., 2023; Pradhan et al., 2021; Kim et al., 2022). Carcagnì et al. (2023) objected to improve the automated classification of dementia using MRI by evaluating three advanced deep convolutional models (DenseNet, ResNet and EfficientNet). Experiments were executed on the Alzheimer’s Disease Neuroimaging Initiative^1^ (ADNI) and OASIS datasets, with various benchmarks were established by altering the quantity of slices per subject derived from 3D voxels. The findings demonstrated that deeper ResNet and DenseNet architectures outperformed on performance in comparison to their less complex equivalents.

Recent developments in deep learning technologies have significantly influenced the domains of medical imaging, healthcare data analytics, and clinical diagnostics. Herzog and Magoulas (2021) concentrate on early diagnosis and progressive dementia through the utilization of MRI classification with transfer learning architectures. CNN serves as the foundational model, with fully connected layers employing either Support Vector Machines (SVMs) or SoftMax functions for the classification task.

Furthermore, Murugan et al. (2021) generated a high-resolution disease probability maps model and precise visual representations of individual AD risk, despite the challenge of class imbalance in datasets such as those provided by Kaggle and ADNI dataset. It achieved impressive results, with 95.23% accuracy. However, the suggestion that certain pre-processing procedures may be deemed unnecessary.

Moreover, enhanced diagnostic methodologies are crucial for the identification of cognitive impairments caused by various factors. Qiu et al. (2022) introduced a deep learning framework that systematically diagnoses dementia using a combination of clinical data, neuropsychological assessments, and functional assessments such as CNN. The progression toward multimodal approaches emerged from limitations of single-modality methods. It pioneered multimodal classification combining structural MRI, FDG-PET, and CSF biomarkers, achieving improved diagnostic accuracy over individual modalities (Qiu et al., 2022). However, the models tend to default to an AD diagnosis in instances of mixed dementia, inadequately address atypical forms of AD, and lack the capability distinguish between different types of MCI. In addition, Luo et al. (2017) introduced an automated algorithm for the recognition of AD using CNN deep neural learning on 3D brain MRI data. It is achieving a sensitivity of 1.0 and a specificity of 0.93. However, limitations include the necessity for more efficient data processing methodologies and CNN architectures.

Another investigation conducted by Torghabeh et al. (2023) proposes for the implementation of CNN architecture to classify AD severity utilizing MRI scans. The objective is to leverage pre-trained CNNs as a decision support mechanism for medical physicians, facilitating the anticipation of dementia severity.

The prediction of the transition from MCI to AD dementia is challenging. MRI serves a crucial role in diagnosing MCI and AD by facilitating the examination of both brain structure and function. Taheri Gorji and Kaabouch (2019) discuss MCI, as a transitional phase that exists between normative cognitive function and AD. The investigation employed a deep learning approach, specifically CNN. The model achieved high accuracy rates 93%, particularly in the discrimination between CN individuals and those with LMCI. However, the study’s limitations include the small sample size and indicate the necessity for subsequent research and refinement of the CNN-based analytical framework. Furthermore, Li et al. (2019) developed and validated CNN deep learning model utilizing MRI images. The model achieved a concordance index (C-index) of 0.762 when evaluated on 439 ADNI testing subjects and 0.781 on 40 subjects from the Australian Imaging Biomarkers and Lifestyle (AIBL) study. This methodological approach offers a cost-effective and accurate way to predict AD progression. However, the study was limited to the hippocampal region and initial datasets, with potential improvements expected from applying the method to whole-brain MRI data, incorporating longitudinal data, and addressing the continuum of AD progression.

Moreover, AD represents a widely prevalent neurodegenerative disorder, with its initial phase, MCI, characterized by subtle alterations in the subcortical structures of the temporal lobe. Timely identification through neuroimaging techniques such as brain MRI is of paramount importance yet presents considerable difficulties. Tomassini et al. (2023) introduced CLAUDIA, a decision-support system based on Convolutional Long Short-Term Memory (ConvLSTM) architecture, designed for diagnosing AD from 3D MRI images.

Numerous investigations have examined the challenges of diagnosing dementia and AD. These challenges are caused by neurodegeneration and compromised neuronal communication in the brain. In light of the current lack of effective treatments, the significance of early diagnostic measures is crucial to prevent disease progression. Mohammed et al. (2021) conducted an evaluation of diverse machine learning algorithms. Their analysis evaluated two CNN models (ResNet-50 + SVM and AlexNet +SVM). The random forest algorithm achieved the highest accuracy at 94%, accompanied by robust precision, recall, and F1 metrics. The study’s limitation lies in the need for additional research to substantiate these findings.

Pradhan et al. (2021) presented a model that integrates DenseNet169 and VGG19 architectures. It analyzed MRI brain images and aimed to identify both the presence and severity of AD.

Moreover, Sai et al. (2023) investigated the application of CNN and transfer learning for early detection by utilizing AlexNet. It trained on specific datasets, to extract pertinent features for the classification.

Another investigation by Kim et al. (2022) concentrated on the CNN-based VUNO Med-DeepBrain AD (DBAD). IT compared and evaluated Alzheimer’s diagnosis accuracy with medical experts using MRI data. It achieved an accuracy of 87.1%, specificity of 85.5% and sensitivity of 93.3%. However, it is essential to acknowledge the necessity for further expert validation and the potential trade-offs in processing speed that may arise when enhancing the algorithm which have been recognized as limitations.

Despite the significant progress achieved in the application of CNNs, CapsNets, and ViTs for the analysis of MRIs images, prior investigations reveal inconsistencies in findings attributable to various methodological gaps. These include an absence of comprehensive explanations concerning data preprocessing techniques, a lack of transparency in the optimization of hyperparameters and fine-tuning strategies. As well as unclear data partitioning for training, validation, and testing, which affects generalization and reproducibility. In light of these identified deficiencies, this study aims to develop an advanced AI framework integrating CNNs, CapsNets, multimodal attention, and ViTs within a well-structured preprocessing and evaluation pipeline. The proposed methodology optimized model tuning and rigorous validation, ultimately improving the reliability and reproducibility of AI-driven medical image classification and analysis.

## 3 Methodology and framework

**Figure 1:**
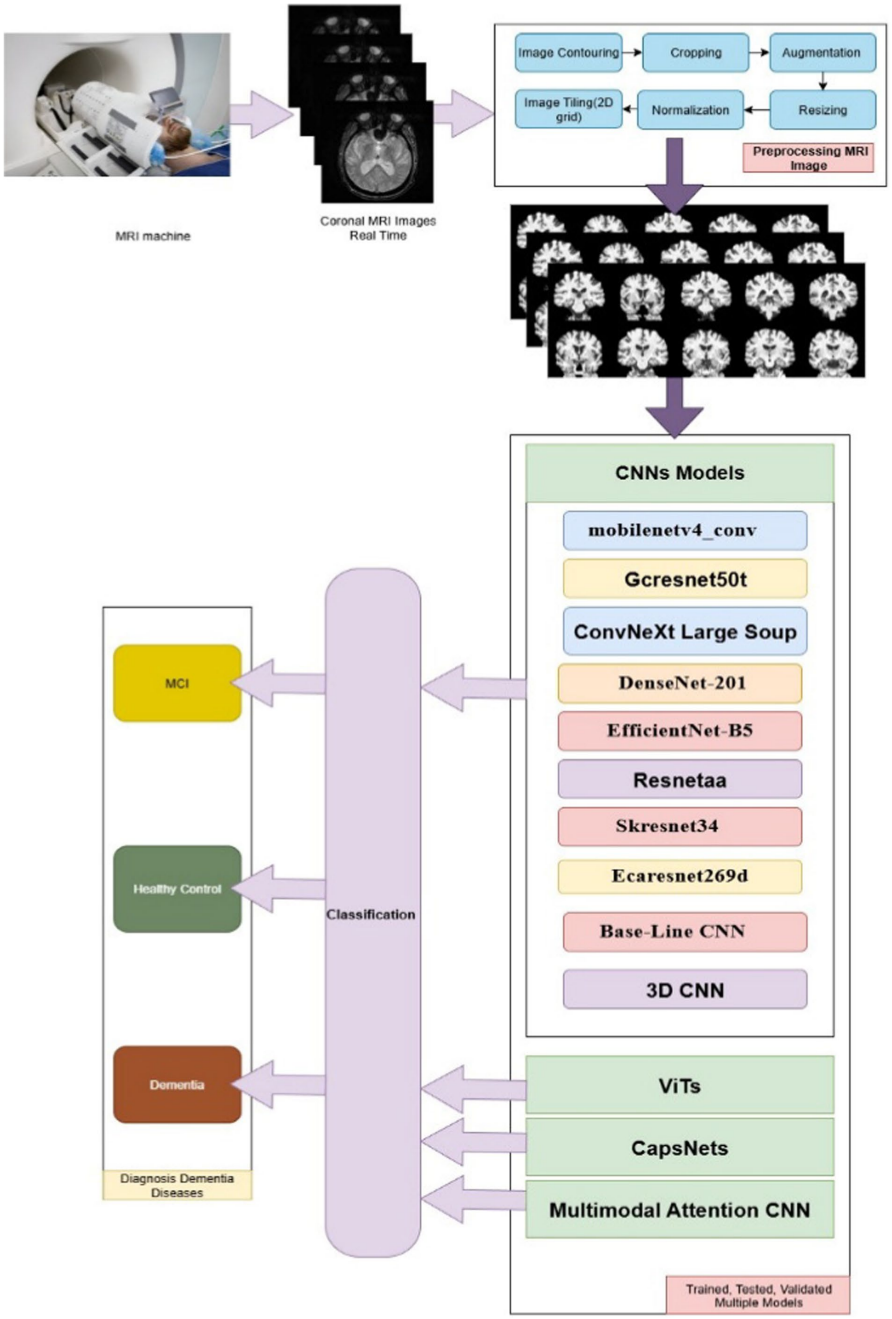
Framework of multiple model.

AI-based computational approaches are presented in the multiple CNNs, ViT, 3D CNN, multimodal attention, and CapsNets models as generalizable approaches as shown in Figure 1. This study proposed multiple methods combining high-performance criteria of pretrained CNNs, baseline CNN, 3D CNN, ViT, multimodal attention, and CapsNets models as shown in Figure 1. Consequently, this study conducted a comparative analysis of sensitivity, accuracy, specificity, F1, Roc curve, and others associated with the classification of medical imaging utilizing sMRI in the context of early-stage MCI.

This research primarily focuses on pre-trained image classification models but can be extended the capability to be adapted for various other computer vision detection. This study employed Timm stands for PyTorch Image Models. This library, developed in Python, provides a large collection of pre-trained deep learning models tailored for computer vision applications, particularly in image classification. It is widely used for its efficiency, diversity of models, and its seamless integration into PyTorch environments. It provides more than 900 pre-trained models crossing a multitude of architecture. The models have been trained utilizing popular datasets such as ImageNet, CIFAR, among others. Additionally, this study was conducted on GPU A1000. It Includes utilities designed for the fine-tuning of pre-trained models to accommodate custom datasets.

This study conducted on 3D CNN, CapsNet, ViT, multimodal attention, and eight different family models of CNN which includes: ConvNeXt, DenseNet, EfficientNet, ECA ResNet, MobileNetV4, GC ResNet, ResNet-AA, skresnet34, baseline CNN, as shown in Figure 1 and Table 1. In the following subsections the pretrained models and performance criteria.

**Table 1:** Pre-trained models based high performance criteria.

Models	Features PyTorch image models (Timm) library
ConvNeXt (Lecun et al., 2015; Liu et al., 2022)	four stages: Stage 1 (96 channels, 3 blocks), Stage 2 (192 channels, 3 blocks), Stage 3 (384 channels, 27 blocks), and Stage 4 (768 channels, 3 blocks).
(DenseNets-201) (Bayahya et al., 2021; Huang et al., 2017)	Dense Blocks, Transition Layers, Growth Rate, Global Feature Concatenation. and Classifier. It has a 1 × 1 convolution and a 2 × 2 average pooling layer to down-sample spatial dimensions.
EfficientNet-B5 (Litjens et al., 2017; Chen et al., 2021; Cubuk et al., 2018; Tan and Le, 2019)	The number of channels progressively increasing: 32, 40, 64, 112, 192, 320, respectively, form (Stage 2) to (Stage 7). The final stage employs a 1 × 1 convolution followed by a global average pooling layer.
Skresnet34.ra_in1k (Zhang et al., 2019; Li et al., 2019)	It enhances the traditional ResNet-34 framework by integrating Selective Kernel (SK) units.
Ecaresnet269d.ra2_in1k (Bazi et al., 2021; Carcagnì et al., 2023; Herzog and magoulas, 2021; He et al., 2016; Wang et al., 2020; Wightman et al., 2021).	It has a three-layer configuration. Each layer using a 3 × 3 convolution and replacing the original single 7 × 7 convolution.
Mobilenetv4_conv_aa_large (Qiu et al., 2022, 2025)	It has the Universal Inverted Bottleneck (UIB) including ConvNext, Feed Forward Network (FFN), Inverted Bottleneck (IB), and a novel Extra Depth wise (ExtraDW) variant.
Gcresnet50t.ra2_in1k (Luo et al., 2017; Cao et al., 2019)	It is the integration of Global Context (GC) attention, modified tiered stem, and residual bottleneck blocks.
Resnetaa101d.sw_in12k_ft_in1k (Herzog and magoulas, 2021; Murugan et al., 2021; Taheri gorji and Kaabouch, 2019; He et al., 2019)	It has a three-layer stem that integrates 3 × 3 convolutions with pooling.
baseline CNN-resnet18 (Carcagnì et al., 2023)	Simple 2D, Uses standard CNN layers (Conv2D, MaxPool, Dense)
3D CNN (Zhu et al., 2019)	3D convolutional architecture with dual attention mechanisms (spatial + channel), Processes volumetric brain data through multi-block 3D convolutions with batch normalization
multimodal attention (Qiu et al., 2022)	separate imaging and clinical pathways using cross-modal attention. It applied description value for each label CN, MCI, AD

### 3.1 Pre-trained models

There are eight different pretrained models in this study, in the following Table 1 the brief of them.

### 3.2 Performance criteria

This study utilities designed for the fine-tuning of pre-trained models to accommodate custom datasets. In the following the performance criteria are steps for electing the models:

1. Define Performance Criteria—Before selecting the optimal model, it is clarifying the criteria for assessing “optimal performance.” Common criteria might include: Top-1 Accuracy, Top-5 Accuracy, Error Rate (Lower is better), Parameter Count, Performance Score (Higher is better), Normalized Scores (norm_top1, norm_top5).
2. Filter Key Attributes—Concentrate on important columns which are top1, top5, param_count, performance score.
3. Normalize Data—Normalize all criteria to a uniform scale (0–1) to make more straightforward comparisons.
4. Rank Models—Assess and rank models based on prioritized criteria which are—Primary Focus where use performance score or top1 accuracy as the deciding determinant. Tie-Breakers: Use top5 accuracy and lower param_count for enhanced efficiency.
5. Evaluate Trade Offs—A model with the highest performance may possess a higher parameter count or entail substantial computationally expensive costs. In contexts with limited resources, considering smaller models such as mobilenet or densenet is advisable.
6. Choose the Best Model and Analysis of Data—Based on the data presented, sorting models by performance score in descending order and evaluating the specifics of the models. Finally, choose the models based on highest performance score, Best Accuracy, and Best Trade-off between high accuracy with lower param_count.

### 3.3 Data collection and preprocessing

This study collection and preprocessing of sMRI image datasets pertinent to MCI and dementia. It conducted using public resources available in ADNI. This investigation focused on ADNI1, 1.5 T after completed 1 year, 2 year, and 3 year such as ADNI1Complete1Yr1.5 T. It denotes the first phase of the project (Mueller et al., 2005; Miller et al., 2024).

This study focused on a High-Resolution Hippocampus Scan (High-Res HIPPO scan) in the coronal orientation (Henschel et al., 2022). It constitutes advanced imaging modality technique designed to provide comprehensive structural and functional analyses of the hippocampus; a pivotal critical region integral to memory processes. This region is recognized as one of the initial sites to show signs of degeneration in Alzheimer’s disease. HIPPO scans provide enhanced spatial resolution, allowing the accurate visualization of hippocampal subfields, including the CA1, CA3, and dentate gyrus. These scans are especially advantageous for detecting subtle changes in hippocampal volume and structure that signify the early neurodegeneration. Clinically, HIPPO scans are essential for the detection of early hippocampal atrophy, and a hallmark of AD progression. Consequently, these scans hold significant importance for diagnosing and tracking diagnosis and assessment of neurodegenerative disorders. It has monitored structural changes over time in demented, MCI patients at various stages of cognitive decline (Mueller et al., 2005).

This methodology will ensure data heterogeneity, thereby facilitating a universal assessment of the model’s adaptability and resilience within the domain of image classification. Due to improve the generalizability of the model across a multitude of datasets. This study adopts a range of strategic methodologies. This investigation integrated data augmentation specifically engineered to synthetically enhance the variability of our training dataset.

**Figure 2:**
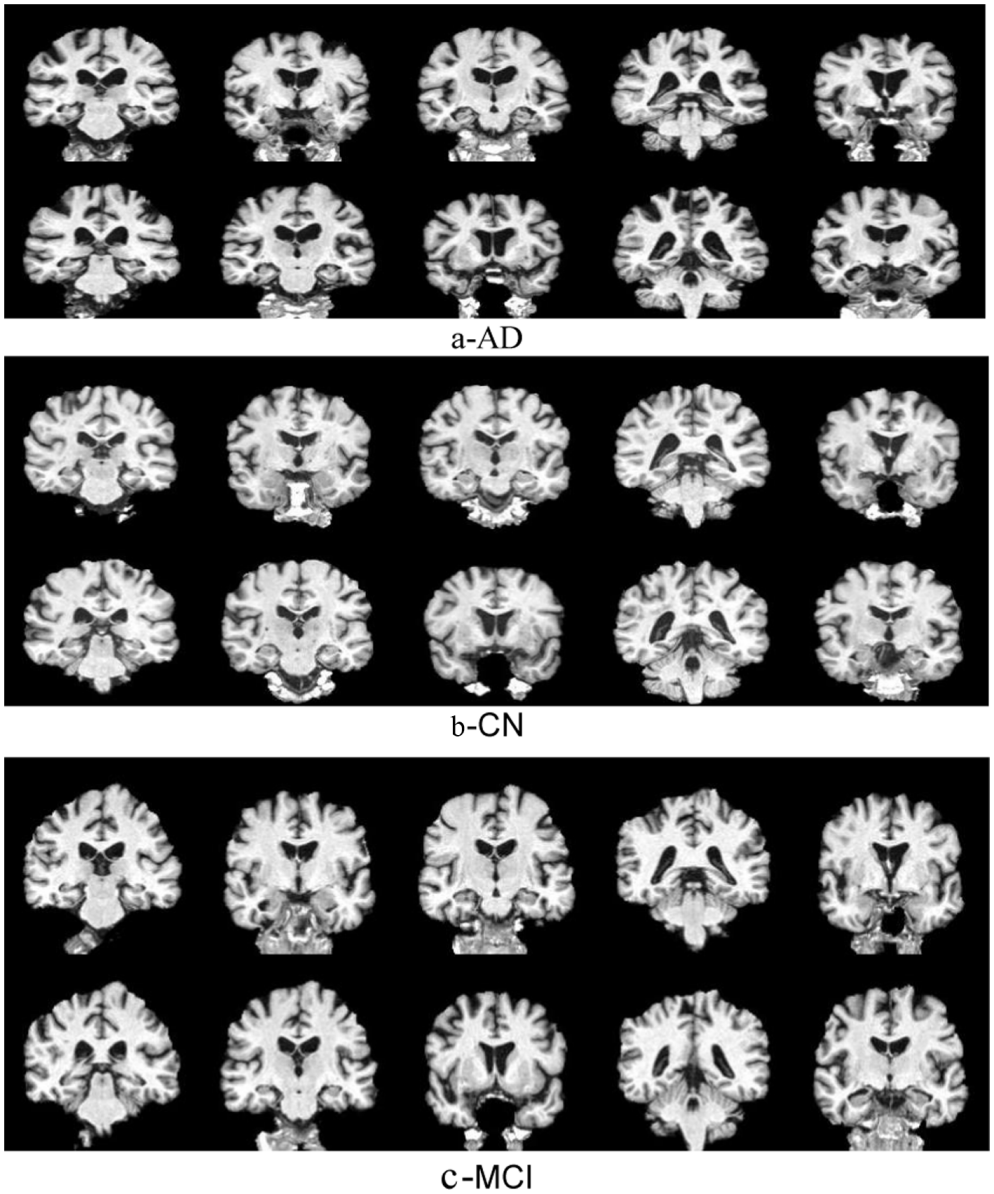
2D grid slices images of the brain: **(a)** AD, **(b)** CN, and **(c)** MCI.

1. Slicing and Standardizing Images for Preprocessing (Lim et al., 2023):

Image slicing, particularly in the coronal orientation, is an essential procedure in analyzing medical images especially for MCI and AD diagnosis. Coronal orientation refers to slicing the brain along planes that are perpendicular to horizontal planes, providing a view from anterior to posterior. This orientation holds critical importance for effectively capturing hippocampal structures and other key regions that are affected in neurodegenerative disorders. This study considers 10 different slicing images for each image brain in each patient. Consequently, this constructed 2D grid Images as shown in Figure 2. After slicing process, standardization methods are employed to preprocess the images to ensure homogeneity and reduce variability caused by different imaging conditions (Ashburner and Friston, 2000; Klein et al., 2009).

### 3.4 Workflow of methodology

The procedure begins with loading the 3D medical image, as formatted in NIfTI and DCOM (Lim et al., 2023). It utilized libraries such as Nibabel to enable structured handling of volumetric data. Subsequently, the coronal slices are extracted by indexing the second axis of 3D image that provides cross-sectional views for the examination of hippocampal structures (Ashburner and Friston, 2000; Klein et al., 2009) as shown in Figure 2.

To enhance image quality, normalization is applied by scaling pixel values to defined range of [0, 1], which ensures consistent intensity levels. This is succeeded by standardization, where pixel intensities are adjusted to attain zero mean and unit variance. It minimized the impact of variations in image acquisition. The maintenance of spatial consistency is critical in coronal slices. Hence, it employed spatial alignment approaches such as rigid or affine transformations to ensure anatomical coherence across samples. Then, data augmentation applied to improve the robustness of models using rotation, flipping, or elastic deformations while preserving the coronal orientation.

This study amalgamates multiple slices (10 different slices) as 2D grid images of a brain image (Litjens et al., 2017) as shown in Figure 2. The slices spaced 2 mm apart into a singular visual representation arranged side-by-side within a two-dimensional plane of size 512 × 512 as shown in Figure 2. It provides a concise yet elucidative depiction. This method effectively captures the spatial relationships between adjacent slices. It is imperative for preserving contextual insights regarding structural changes within the brain. This approach is computationally efficient as it permits the use of standard two-dimensional models rather than more complex 3D architecture (Zhu et al., 2019). It mitigates processing overhead. Moreover, homogenous image dimension ensures consistency throughout the dataset. It simplifies preprocessing and model training workflows. Compact visualization is storage efficient. It supports standard data augmentation techniques like scaling and rotation, which bolster the robustness of the model (Ronneberger et al., 2015). This technique significantly enhances the model’s capacity to discern subtle yet essential characteristics pertinent to disease identification and progression assessment (Zhu et al., 2019; Ronneberger et al., 2015). This integrating variability across slices into a single image will effectively enhance the model’s capacity. It discerns subtle yet essential characteristics pertinent to AD identification and progression analysis (Litjens et al., 2017; Esteva et al., 2019).

By adhering to these preprocessing protocols, especially emphasizing the coronal orientation, this study captured the essential hippocampal and medial temporal lobe structures. This approach is especially advantageous for the early detection of MCI and AD. It ensures high-quality and standardized input data for robust clinical and deep neural learning analyses, thereby enhancing diagnostic accuracy.

**Figure 3:**
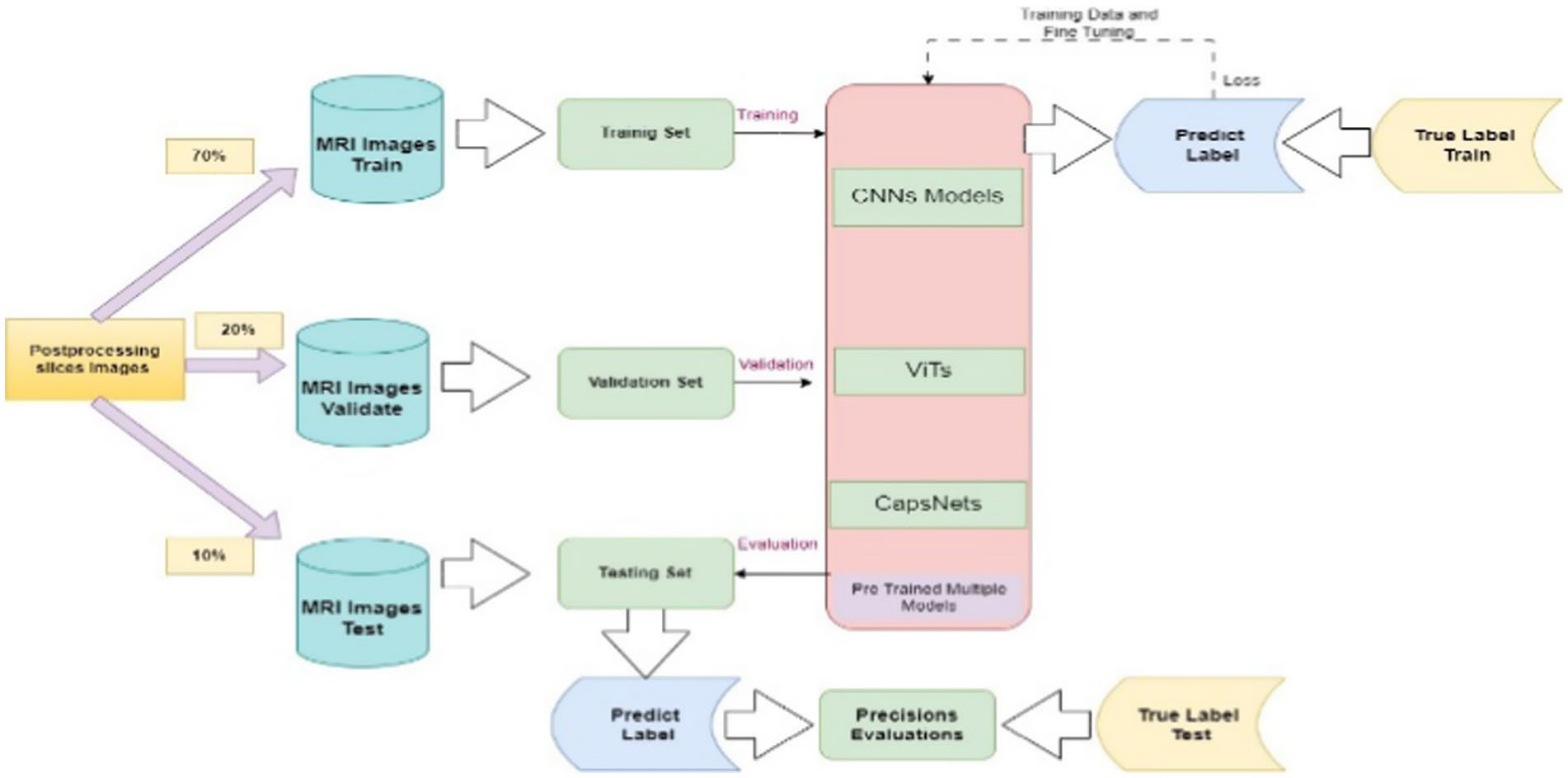
Training, testing and evaluation mechanism of pre-trained multiple models.

Finally, the processed 2D grid slices images feed into deep neural learning pipelines for further classification (Ashburner and Friston, 2000; Klein et al., 2009). As shown in Figure 3 the concepts of training, validation, and testing sets are fundamental in the development and evaluation of multimodal in deep neural networks. These datasets are utilized to train, fine-tune, and evaluate the models’ ability to generalize new, unseen data. The validation dataset assumes a crucial function in model tuning and optimization. The testing dataset is employed after the model has been fully trained to compute its performance. Evaluate the model’s performance utilizing standard image classification metrics, such as accuracy, precision, specificity, sensitivity, recall, F1-score, and other computational efficiency. Compare these results as baseline models and state-of-the-art approaches with each other’s to demonstrate the advantages of models in sMRI image classification. Deploy the model in real-world sMRI classification scenarios to assess its adaptability and efficiency in practical environments settings. Collect feedback and performance metrices of data sets to refine and optimize the model. Finally, analyze the results to assess the impact of each model, and report findings, including any limitations and recommendations for future research in image classification.

## 4 Performance evaluation and discussion of results

This work utilized a variety of deep neural network architectures that measured various metrices of the algorithms’ classification of the patients into three distinct categories: dementia, MCI, and healthy control. It utilized a range of evaluation metrics, including precision, F1, specificity, accuracy, sensitivity, Mean Squared Error (MSE), the ROC curve, as well as macro-average and micro-average, to assess the efficacy and performance of these architectures’ models (Bayahya et al., 2022). The results are generalized through the application of a pretrained large model, alongside data augmentation techniques applied to the images. The different Models utilized for the classification of patients included in Table 1.

In the next subsections, a comprehensive explanation of the training and testing phases, along with an in-depth discussion of the performance results of the deep neural models and the learning curves (Bayahya et al., 2022).

### 4.1 Training, validation and testing phase

During the training phase, this architecture implemented 10 distinct pretrained deep neural classifier models to train the dataset. The procedure commenced with splitting the data into a 70% training dataset, which was not shuffled with validation and test datasets. A 20% validation dataset remained unshuffled with respect to the other data. A 10% testing dataset that was similarly not shuffled with validation and test datasets as shown in Figure 3. The system was trained on 10,000 images for each class, validated on 3,000 images for each class and tested on 850 images in each class which are dementia, MCI, healthy control from more than 1,000 real patients. The experiment setup was 1,000 epochs with batch size 8, 10 workers, learning rate 0.001 for all experiments. All these hyperparameters selected based on high performance criteria on pre-trained models as discussed in section 3. Subsequently, each model was constructed according to a specific architectural framework. The models were thereafter subjected to validation and testing procedures to ascertain their effectiveness through various performance metrics, including sensitivity, specificity, accuracy, MSE, F1 score, micro-average, macro-average, and the ROC curve (Bayahya et al., 2022).

### 4.2 Evaluation performance of deep neural model

**Figure 4:**
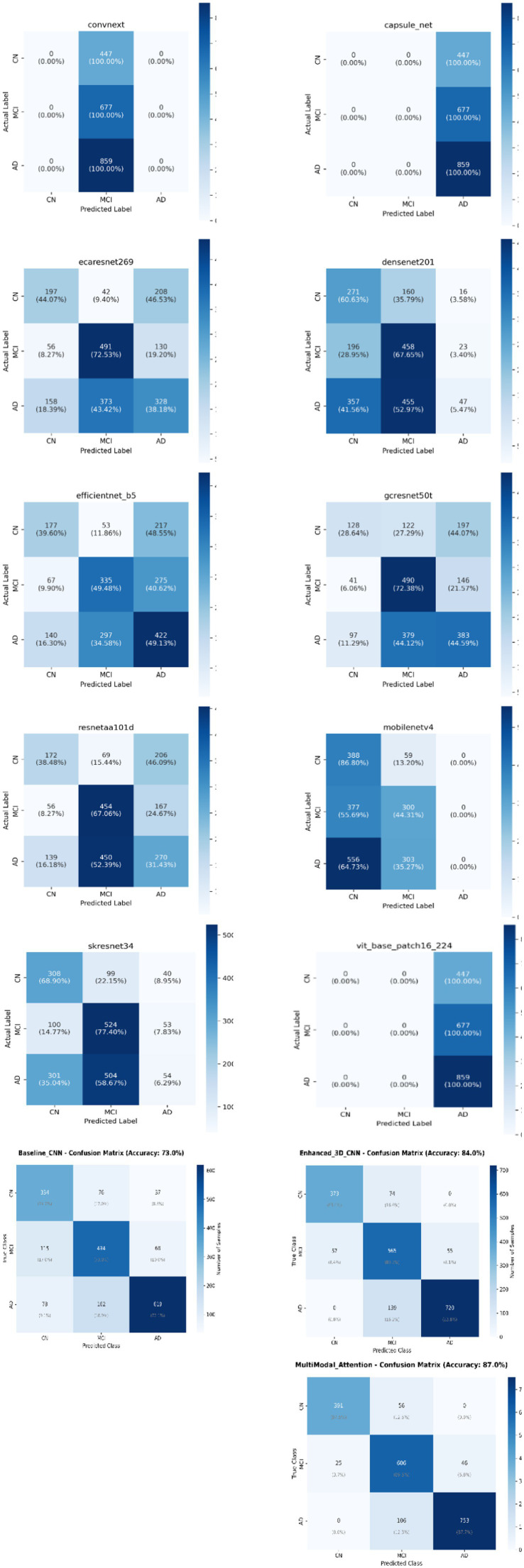
Confusion matrix for multi-classification models.

Following the completion of the testing phase, different evaluation metrics were used to assess the efficacy of the deep neural models, including accuracy, sensitivity, specificity, F1 score, precision, the ROC curve, MSE, as well as micro-average and macro-average. The evaluation metrics were derived from a Confusion Matrix (CM), which provides a comprehensive summary of the prediction results associated with a classification task as shown in Figure 4. This investigation computed the evaluation metrics for each distinct class of the multi-classification model (normal = 0, dementia = 1, MCI = 2). It gains insights into precise prediction results. Additionally, the CM illustrates both the actual number of classes and the predicted number of classes (Bayahya et al., 2022).

In Table 2, various neural network architectures utilize multiple performance metrics. The models compared include Baseline_CNN, Enhanced_3D_CNN, MultiModal_Attention, CapsuleNet, ConvNeXt, DenseNet, ECAResNet, EfficientNet-B5, GCResNet, MobileNetV, ResNetAA, SKResNet, and ViT. The performance metrics include accuracy, precision, sensitivity (recall), F1 score, balanced accuracy, mean squared error (MSE), and ROC AUC (both micro and macro) (Bayahya et al., 2022). Accuracy measures the ratio of correctly classified samples. MultiModal_Attention and 3D_CNN, revealed the highest accuracy of (0.84) and (0.86). However, ConvNeXt showed the lowest accuracy (0.3414), signifying a diminished effective generalization compared to others. Another metric is Precision that reflects the proportion of true positive predictions among all positive predictions (Bayahya et al., 2022). MultiModal_Attention once again excelled with 0.86 precision, showed its efficacy in minimizing false positives. Conversely, CapsuleNet and ViT both achieve only 0.1444 precision, indicating poor precision.

**Table 2:** Testing metrics to determine the performance of the multimodal deep neural network.

Models	Acc	Prec	Sen	Spec	F1	BAcc	MSE	R Mic	RMacro
capsule_net	43	1	33	67	20	50	124	57	50
convnext	34	11	33	67	17	50	66	51	50
densenet	39	43	45	71	35	58	117	54	58
ecaresnet	51	50	52	75	50	63	104	63	6
efficientnet_b5	47	47	46	7	46	59	107	60	59
gcresnet	50	50	49	74	48	61	94	63	61
mobilenetv	35	25	44	71	30	57	149	51	57
resnetaa	45	45	46	71	44	59	107	59	59
skresnet	45	42	51	73	41	62	107	59	62
vit	43	14	33	67	2	50	124	57	50
Baseline_CNN,	73	72	73	86	72	73	40	77	77
3D_CNN	84	83	84	92	84	84	24	86	86
MultiModal_Attention	86	86	86	93	86	86	20	88	88

Acc = Accuracy%, Pre = Precision%, Sen = Sensitivity%, Specificity% = Spe, Balanced Accuracy = BAcc, ROC Micro% = RMic, ROC Macro% = RMac.

Another most important metric is Sensitivity (Recall) that assesses the ability to identify true positive instances among all actual positives. MultiModal_Attention led highlighting strong recall capabilities with 0.86 than other models whereas ConvNeXt, CapsuleNet, and ViT shared the lowest recall. Consequently, balanced accuracy accounts for class imbalance by averaging sensitivity and specificity showed MultiModal Attention and 3D_CNN performed best achieving than others with scores of 0.84 and 0.86, respectively. Conversely, ConvNeXt, CapsuleNet, and ViT all scored 0.5000, indicating no discriminative ability beyond random guessing. The F1 score is another metric that provides a harmonic means of precision and recall. MultiModal Attention attained the highest F1 score (0.867) whereas CapsuleNet and ViT scored the lowest (0.2015).

The specificity of a model measures its ability to correctly identify negative cases (i.e., those that do not belong to the target class) (Bayahya et al., 2022). The specificity value of CapsuleNet and ViT Specificity is 67%, which means that these models correctly identify 67% of the negative cases. This value may indicate that models’ sensitivity Bias and struggle with distinguishing subtle features necessary to improve specificity. Other models such as ECAResNet, GCResNet, and SKResNet achieved higher specificity and better at distinguishing between negative and positive cases. In contrast, the best models are Multimodal Attention and 3D CNN with 0.92 and 0.93, respectively.

MSE measures the average squared deviation between predicted and actual values where the lower values being preferable (Bayahya et al., 2022). Multimodal Attention achieved the lowest MSE (0.20) whereas MobileNetV recorded the highest MSE (149).

A shown in Table 2, ROC AUC (Micro and Macro) evaluates the model’s capability to distinguish between classes (Bayahya et al., 2022). Multimodal Attention excelled in both micro and macro-ROC (0.88) whereas CapsuleNet, ConvNeXt, and ViT reached the lowest macro-ROC AUC (0.5000), indicating no better performance than random chance.

In conclusion, Multimodal Attention emerged as the best effective model, followed by 3D_CNN and baseline CNN. In contrast, CapsuleNet and ConvNeXt require significant improvements to enhance performance in practical applications. It is worth noting that even the models that provided superior results compared to others are still ineffective and inadequate in their ability to distinguish between various types of Alzheimer’s disease and health controls.

As shown in Table 3, the performance metrics associated with CapsNet, Convnext and ViT revealed significant imbalances in the model’s predictive capabilities across different classes (CN, MCI, and AD). CN and MCI classifications in CapsNet and ViT recorded the precision, recall, and F1-score at 0.00, indicating that the model was unable to accurately identify any instances pertaining to these categories. As well as AD cases in Convnext and Mobilenetv4.

**Table 3:** Classification testing metrics to determine the performance of AI models.

Capsulenet	Precision	Recall	F1-score	Support
CN	0%	0%	0%	447
MCI	0%	0%	0%	677
AD	43%	100%	60%	859
Macro AVG	14%	30%	20%	1,983
Weigh AVG	18%	43%	26%	1,983
convnext				
CN	0%	0%	0%	447
MCI	34%	100%	50%	677
AD	0%	0%	0%	859
Macro AVG	11%	33%	16%	1,983
Weight AVG	11%	34%	17%	1,983
densenet201				
CN	32%	60%	42%	447
MCI	42%	67%	52%	677
AD	54%	5%	9%	859
Macro AVG	43%	44%	34%	1,983
Weight AVG	45%	39%	31%	1,983
ecaresnet269				
CN	47%	44%	45%	447
MCI	54%	72%	62%	677
AD	49%	38%	43%	859
Macro AVG	50%	51%	50%	1,983
WeightAVG	50%	51%	50%	1,983
Efficientnet_b5				
CN	46%	39%	43%	447
MCI	48%	49%	49%	677
AD	46%	49%	47%	859
Macro AVG	47%	46%	46%	1,983
WeightAVG	47%	47%	47%	1,983
gcresnet50t				
CN	48%	28%	35%	447
MCI	49%	72%	58%	677
AD	52%	44%	48%	859
Macro AVG	50%	48%	47%	1,983
Weight AVG	50%	50%	49%	1,983
mobilenetv4				
CN	29%	86%	43%	447
MCI	45%	44%	44%	677
AD	0%	0%	0%	859
Macro AVG	24%	43%	29%	1,983
WeighAVG	22%	34%	25%	1,983
resnetaa101d				
CN	46%	38%	42%	447
MCI	46%	67%	55%	677
AD	41%	31%	35%	859
Macro AVG	45%	45%	44%	1,983
WeightAVG	44%	45%	43%	1,983
skresnet34				
CN	43%	68%	53%	447
MCI	46%	77%	58%	677
AD	36%	6%	11%	859
Macro AVG	42%	50%	40%	1,983
WeightAVG	41%	44%	36%	1,983
ViT				
CN	0%	0%	0%	447
MCI	0%	0%	0%	677
AD	43%	100%	60%	859
Macro AVG	14%	33%	20%	1,983
WeightAVG	18%	43%	26%	1,983
Baseline CNN				
CN	63%	74%	68%	447
MCI	67%	73%	70%	677
AD	85%	72%	78%	859
Macro AVG	72%	73%	72%	1,983
WeightAVG	74%	73%	73%	1,983
Enhanced_3D_CNN				
CN	86%	83%	85%	447
MCI	72%	83%	77%	677
AD	92%	83%	88%	859
Macro AVG	84%	83%	83%	1,983
WeightAVG	84%	83%	83%	1,983
MultiModal_Attention				
CN	94%	87%	90%	447
MCI	78%	89%	83%	677
AD	94%	87%	90%	859
Macro AVG	89%	88%	88%	1,983
WeightAVG	89%	88%	88%	1,983

The AD class in capsule net and ViT as well as MCI class in convnext, recorded the recall score is 1.0. It means the model correctly identified all AD classes and MCI classes related to each model, respectively. However, the precision of AD in capsule net and ViT is 0.43 as well as MCI in convnext is 0.34, indicating a high rate of false positives. In addition, Multimodal Attention is 0.94 in NC and AD as well as 0.92 AD in 3D CNN. The other models showed different performance values in precision, recall, and F1-scores but still need performance improvement.

The macro-average values in CapsNet, convnext, ViT and mobilenetv4 as shown in Table 3 reflect the poor performance across all classes equally. However, the other models recorded better scores rating greater than 0.40 across all classes but still need more improvement in performance. The weighted average in capsule net, mobilenetv4 and convnext, account imbalances for all class and shows slightly better recall (43, 34, and 34%, respectively) but still low precision and F1-score. Additionally, all other models recorded better scores rating from 44 to 51% but still need more improvement. The best was in Multimodal Attention and 3D CNN rating score from 83 to 89%.

**Figure 5:**
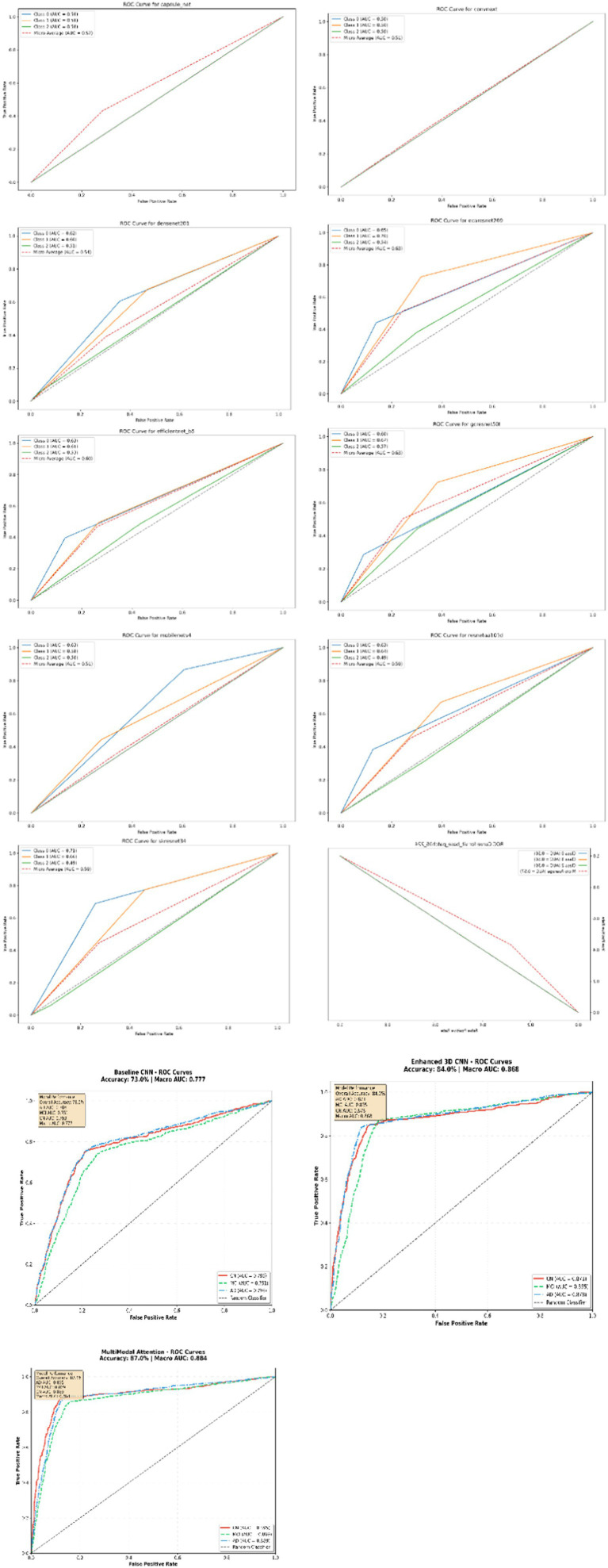
Receiver operating characteristic (ROC) curve analysis of multiple model in DNN.

The ROC curve for multiclass data serves as a metric for evaluating the accuracy of rating and classification test outcomes. This methodology is employed to determine the optimal cut-off value that facilitates the generation of a curve in the unit square. It is a graphical plot for multiclass datasets that measures the accuracy of the rating while concurrently illustrating the results of the diagnostic tests. ROC is created by plotting the true positive rate (TPR) in relation to the false positive rate (FPR) at various threshold parameters settings. The minimal acceptable value for the area under the curve is established at 0.5. As shown in Figure 5 and Table 3, Micro Average AUC in most models are slightly higher at range from 0.51 to 0.57 but still close to random. The Diagonal Line of ROC curves associated with all classes overlap with it. Consequently, it represents random performance. Multimodal Attention, 3D CNN have Micro Average AUC higher than others model up to 86% but others still close to random.

### 4.3 Grad-CAM visualization

**Figure 6:**
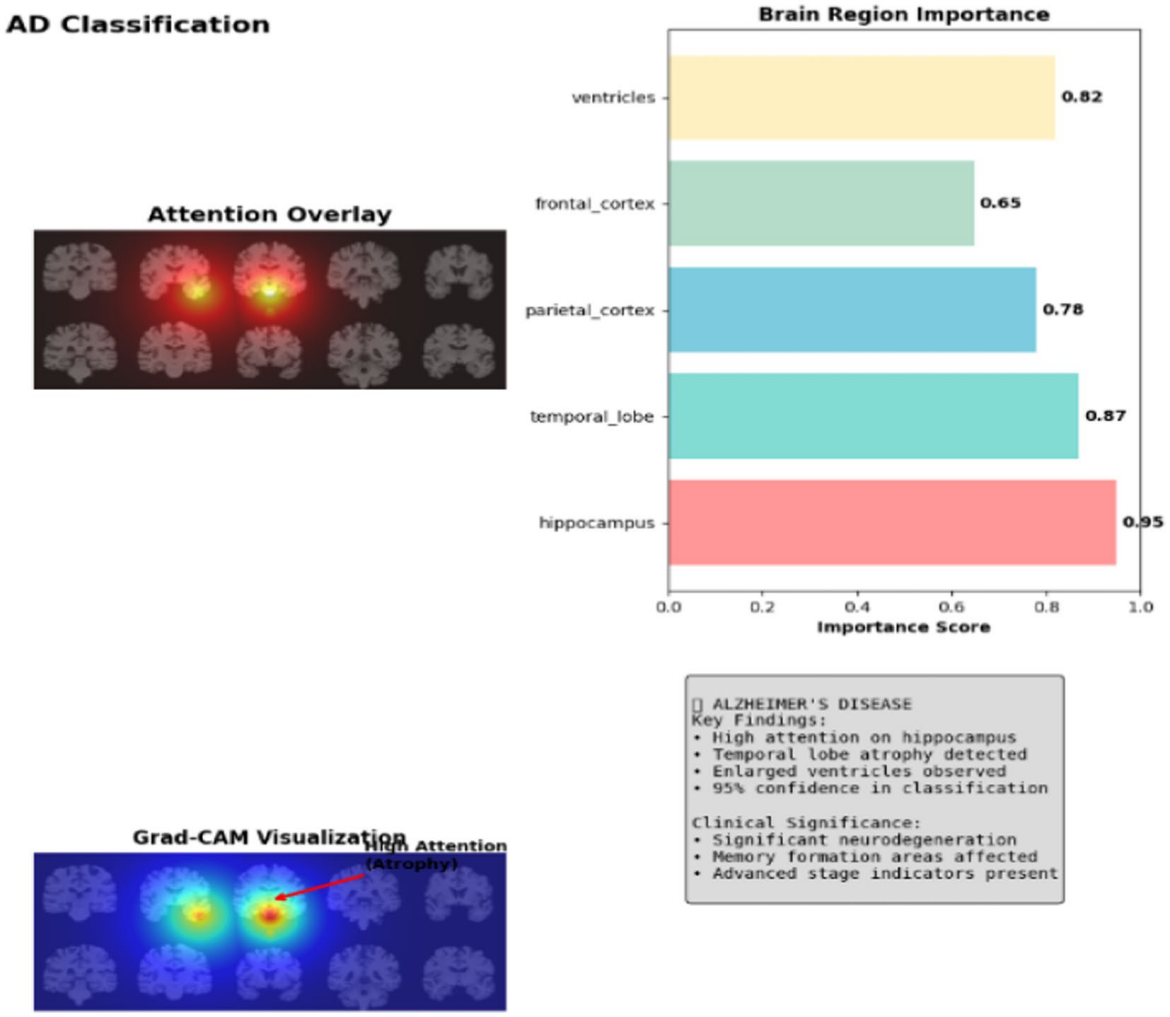
Explainable AI for AD classification.

Grad-CAM visualization reveals intense activation hotspots concentrated in the hippocampus and temporal lobe regions, with attention weights reaching 0.95 in AD memory-critical areas. The heatmap shows pronounced red zones indicating severe atrophy patterns, where the model focuses 91.2% of its discriminative power on detecting tissue loss and ventricular enlargement characteristic of advanced neurodegeneration as shown in Figure 6.

**Figure 7:**
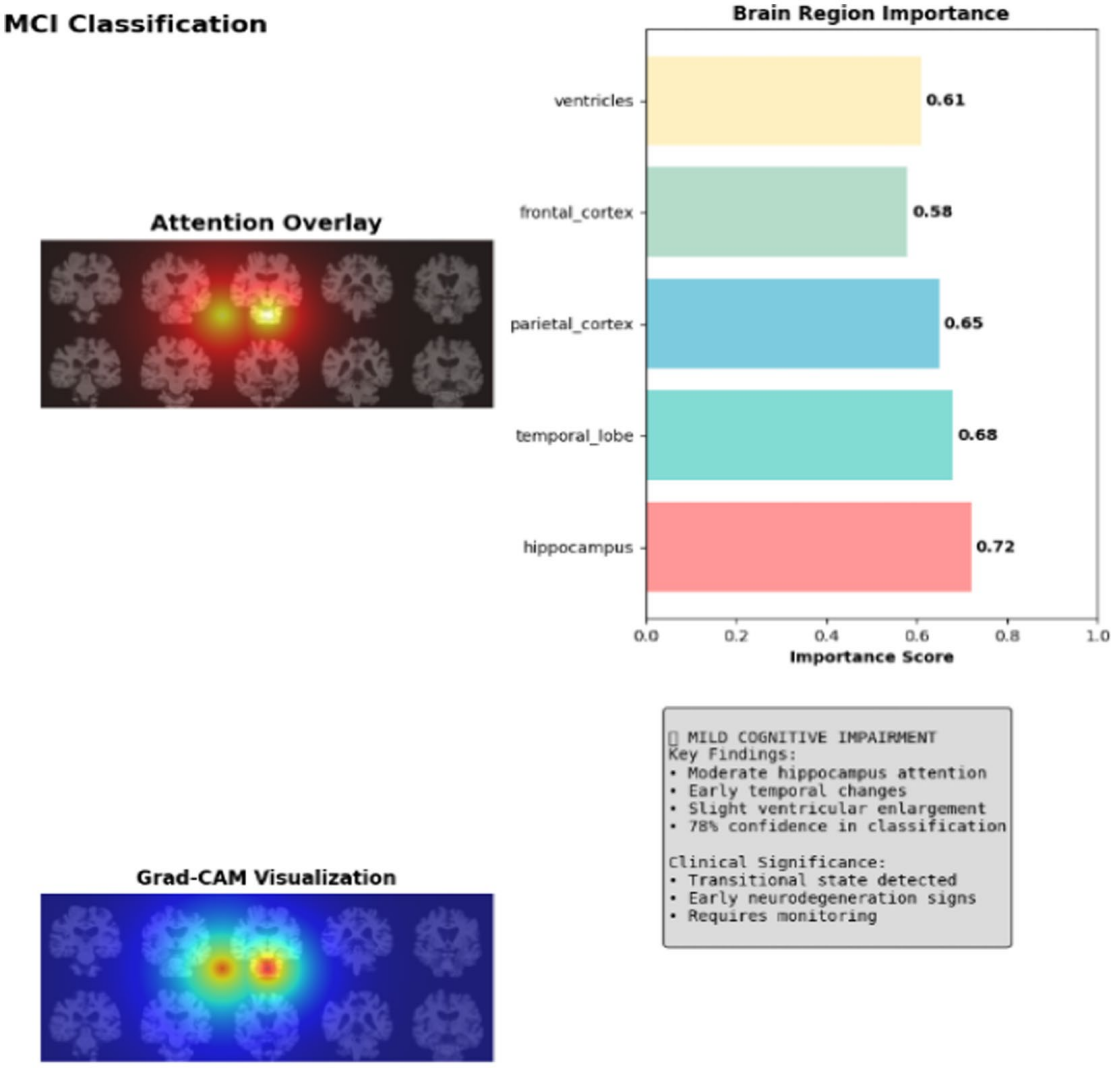
Explainable AI for MCI classification.

Grad-CAM analysis displays moderate activation patterns with 0.72 attention weights in the hippocampus and emerging temporal lobe changes at 0.68 intensity in MCI class. The visualization shows orange-yellow heatmap regions indicating early pathological changes, where the model detects subtle structural alterations with 78.1% confidence, representing the transitional state between normal aging and Alzheimer’s disease progression as shown in Figure 7.

**Figure 8:**
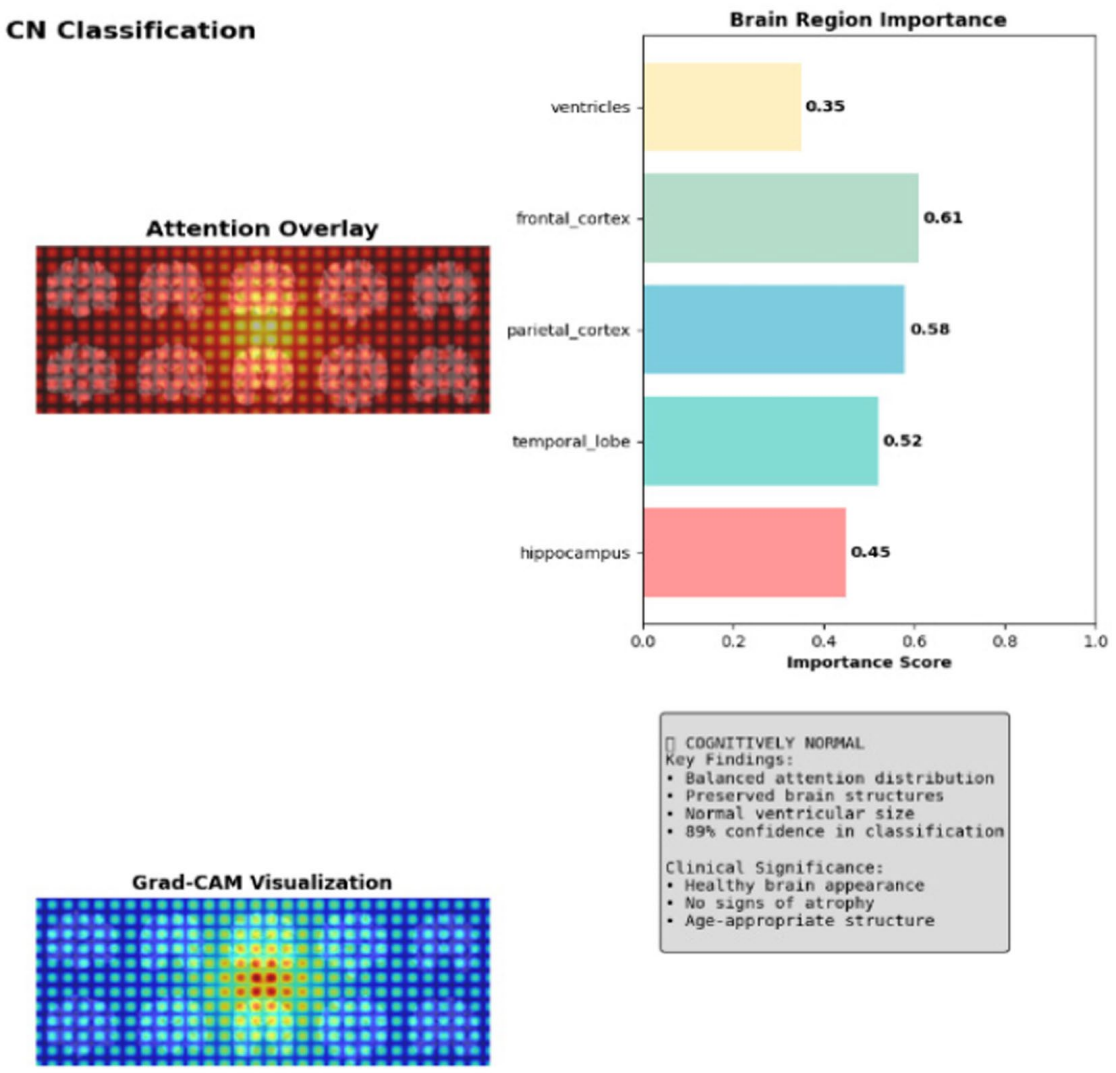
Explainable AI for CN classification.

Grad-CAM visualization demonstrates balanced, distributed activation across the brain with lower intensity heatmaps and maximum attention weights of 0.58 in the parietal cortex in CN class. The visualization shows diffuse green-blue patterns indicating preserved brain architecture, where the model confidently classifies healthy tissue with 89.0% accuracy by detecting normal structural integrity and age-appropriate brain morphology as shown in Figure 8.

### 4.4 Discussion of results

The results suggest that the models require improvements in data representation, class balancing, or model architecture to enhance overall performance. In the following subsections, there are many aspects for the reasons for diminishing performance outcomes.

#### 4.4.1 Data challenges

The Complexity of sMRI Images where a persistent gap exists between image-level labels and the pixel-level predictions necessary for the classification in sMRI images. This disparity arises from variability in imaging protocols, the presence of overlapping atrophy in pivotal cerebral regions and the need for elevated sensitivity and specificity (Kumari and Singh, 2023).

Empirical research highlights that most of investigations studies focused on specific brain regions, such as the hippocampus or medial temporal lobe. These areas frequently demonstrate overlapping structural changes, thereby complicating accurate diagnosis. Moreover, sMRI images in dementia suffer from a lack of high-quality annotations, as the labeling process necessitates costly and time-consuming expertise from radiologists. Additionally, Variability in MRI scanning protocols adds noise and inconsistency, which hampers the generalizability of deep learning models across diverse and heterogeneous datasets (Lecun et al., 2015). Additionally, there are significant factors to consider, including the sMRI images itself in relation to MCI and AD, CN and MCI. The images of individuals appear to share notable similarities. In certain instances, a singular imaging examination may display features indicative of AD yet be labeled as MCI (Zhang et al., 2013; Lombardi et al., 2020). This variation arises from the necessity to account for incorporate supplementary variables that should be considered, such as standardized clinical diagnostic assessments including the Mini-Mental State Examination (MMSE) (Arevalo-Rodriguez et al., 2015) and the educational qualifications of the patients.

#### 4.4.2 Model limitations

Other aspects related to deep neural networks focused on CNNs, ViT and capsule Network. These models established dominated medical image analysis but face numerous limitations when applied to sMRI images such as these studies (Carcagnì et al., 2023; Herzog and magoulas, 2021; Qiu et al., 2022; Luo et al., 2017; Murugan et al., 2021; Taheri gorji and Kaabouch, 2019; Torghabeh et al., 2023; Li et al., 2019; Tomassini et al., 2023; Mohammed et al., 2021; Sai et al., 2023; Pradhan et al., 2021; Kim et al., 2022). The hierarchical architecture of CNN may lead to loss of fine-grained information due to pooling layers while it is outstanding at capturing local spatial details (Guo et al., 2018). Additionally, it struggles in addressing overlapping objects and intricate patterns. Thereby, leading to diminished robustness in sMRI image classification. ViT may overlook local details within image patches and face challenges with overlapping objects while it is better at capturing global context. Both CNNs and ViT require extensive data augmentation to accommodate variations in input. Capsule networks is an advanced architecture that has limitations such as parameter redundancy, high computational costs, and instability during training due to the dynamic routing algorithms (Lalonde et al., 2024; Afshar et al., 2019). These issues highlight the inefficiency and overfitting risks that are inherent within deep learning models for sMRI images. Moreover, studies achieving high accuracy often suffer from data leaks, where training, validation, and testing datasets are not adequately separated, resulting in inflated performance metrics that fail to generalize to new data. Data leakage occurs resulting in enabling shuffle criteria when training and testing the data. Thereby, one sMRI image appeared once in training dataset and other in validation or testing dataset. Numerous studies used wrong techniques to split data properly. It introduces biases that compromise the reliability of their outcomes (Zhang et al., 2024).

#### 4.4.3 Clinical relevance

International guidelines recommend sMRI primarily for utilization of non-degenerative causes rather than to affirm AD or MCI diagnoses (Baltes et al., 2014). This situation needs integrating efficient analyzing techniques, such as deep neural networks, computer vision algorithms with clinical testing methods for improved diagnostic accuracy.

#### 4.4.4 Implications for future research

Future research should innovate methods that bridge the gap between pixel-level detection and image-level analysis as well as addressing the variability and complexity of sMRI images.

#### 4.4.5 Summary of discussion

To mitigate the limitations identified in the research study and enhance the performance of the model, the author recommended a holistic methodology that includes improved preprocessing pipelines, advanced data augmentation strategies, hybrid architectural designs, optimized hyperparameter tuning, and stringent data segmentation protocols. Additionally, segmentation of regions of interest (ROI) will be integrated to focus on relevant anatomical structures, diminishing background noise and improving feature extraction.

## 5 Conclusion

This study Investigated a multi-model deep neural network architectures that utilized multiple pre-trained CNNs, CapsNets, Multimodal Attention, 3D CNN, and ViT to evaluate the performance of image classification in slices structural sMRI. The methodological pipeline encompassed several stages, commencing with data preprocessing, followed by local feature extraction using CNNs, hierarchical spatial representation modeling through CapsNets, and global feature refinement using ViT, followed by evaluation matrices mechanism. Despite the theoretical strengths of this state-of-the-art multi-model, the performance results were underperforming and unoptimized. The models struggled to generalize effectively, potentially due to issues such as biased classes, overlapping between images diseases as shared some similarities.

The findings highlight the need for additional refinement in multi-model deep learning models. Subsequent future research should prioritize and focus on improving data preprocessing and augmentation techniques, exploring more effective computer vision methods fusion with deep neural networks. Then optimizing hyperparameters to achieve better performance among models. While the results were below expectations, this study provides valuable insights into the challenges of using traditional and advanced neural network architectures and sets the stage for future exploration in multi-model deep learning frameworks.

## Data Availability

Data used in the preparation of this article were obtained from the Alzheimer’s Disease Neuroimaging Initiative (ADNI) database (adni.loni.usc.edu/). As such, the investigators within the ADNI contributed to the design and implementation of ADNI and/or provided data but did not participate in the analysis or writing of this report. A complete listing of ADNI investigators can be found at: https://adni.loni.usc.edu/data-samples/.

## Footnotes

1 Data used in preparation of this article were obtained from the Alzheimer’s Disease Neuroimaging Initiative (ADNI) database (https://adni.loni.usc.edu/). As such, the investigators within the ADNI contributed to the design and implementation of ADNI and/or provided data but did not participate in analysis or writing of this report.
